# Redo TAVI: Improved Hemodynamics With a Supra-Annular Valve in a Small Annulus

**DOI:** 10.1016/j.jaccas.2025.106501

**Published:** 2025-12-17

**Authors:** Alok D. Shah, Anwar Hussain, Andrew Wiper, David Roberts, Rebecca Jones, Amr Gamal, Hesham Abdelaziz, Ranjit More, Tawfiq Choudhury

**Affiliations:** Blackpool Teaching Hospitals NHS FT, Blackpool, United Kingdom

**Keywords:** aortic valve, stenosis, valve replacement

## Abstract

**Background:**

Expanding transcatheter aortic valve implantation (TAVI) indications to include severe aortic stenosis patients at a younger age, combined with increasing patient survival, have led to a rise in Redo TAVI procedures. In them, patients with a small native annulus pose a unique challenge.

**Case Summary:**

We describe such a patient who presented with early transcatheter heart valve (THV) failure and underwent redo TAVI with an Allegra THV, with an emphasis on redo TAVI planning concepts & process.

**Discussion:**

The Allegra THV's distinctive design allows for higher effective orifice areas in small annuli while commissural post movement owing to a flexible outflow reduce leaflet stress.

**Take-Home Messages:**

Redo TAVI procedures require meticulous CT analysis for procedural and hemodynamic success. The Redo TAVI app by Dr Bapat is an essential aid, and the Allegra THV a useful consideration in redo TAVI procedures with small annuli.

## Central Figure

**Figure:**
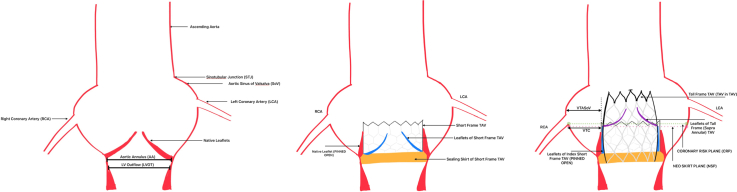

## History of Presentation

An 86-year-old lady presented with increasing breathlessness (NYHA Class III). Examination revealed a third heart sound at apex, an ejection systolic murmur in the aortic area with an early diastolic murmur in the left third intercostal space.

## Past Medical History

She had presented with severe aortic stenosis (AS) in 2021. Annulus measured 336 mm^2^ on CT TAVI and a 20 mm SAPIEN 3 THV (Edwards Lifesciences Corp) was implanted. Subsequently, a dual-chamber pacemaker was implanted for alternating bundle branch block and AV nodal ablation for atrial fibrillation done. Postprocedural echocardiography showed peak gradient (PG) and mean gradient (MG) of 29 and 17 mm Hg, respectively, with LVEF of 60%.

## Differential Diagnosis

Early transcatheter heart valve (THV) failure was deemed more likely in view of gradual symptom progression and absence of signs of infective endocarditis.

## Investigations

Echocardiography showed PG/MG of 46/29 mm Hg with moderate aortic regurgitation (AR), an indexed effective orifice area (iEOA) of 0.70 cm^2^/m^2^, and LVEF of 35%. Transesophageal echocardiography showed severe transvalvular AR. The mechanism of early bioprosthetic valve failure was felt to be thickening and fixation of the noncoronary cusp of the THV (Summary of echocardiographic parameters available in Appendix 1).

Following multi-disciplinary team discussion, review of the repeat CT and considering patient frailty & available surgical expertise, redo transcatheter aortic valve implantation (TAVI) was offered to and accepted by the patient. CT images are shown below (detailed CT measurements and sizing methodology are available in the Appendix 2).

**Figure 1:**
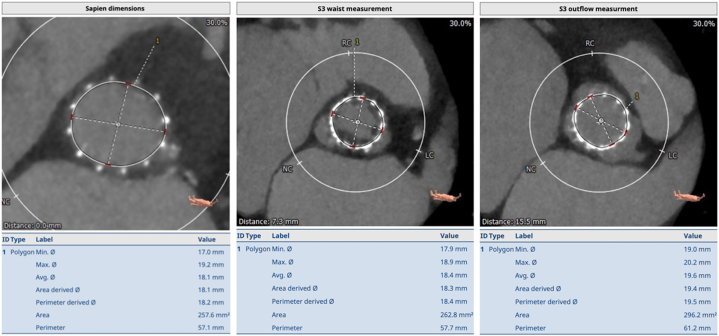
Measurements at Inflow, Waist and Outflow of the Index Transcatheter Aortic Valve (Edwards S3 20)

**Figure 2:**
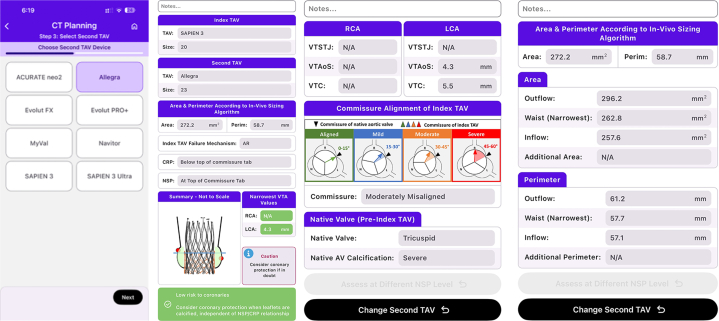
Screenshots of Valve Options and Summary Report Generated by the Redo TAV App (Copyright© Vinayak Bapat)

**Figure 3:**
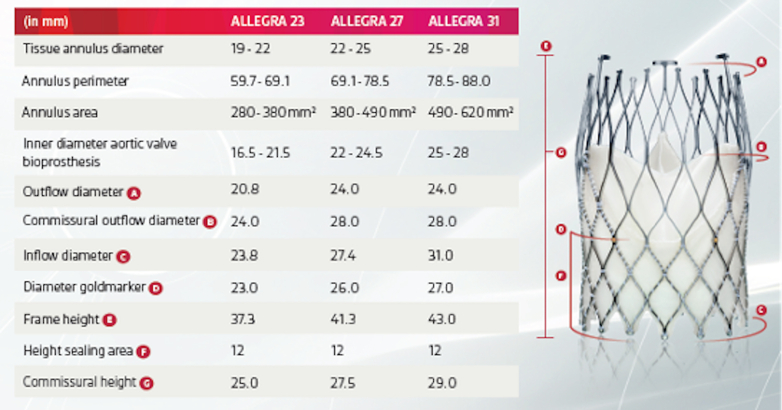
ALLEGRA Valve: Features & Sizing

Due to better hemodynamic performance in small annuli (<430 mm^2^),^1^ a self-expanding supra-annular THV was considered optimal, with the ALLEGRA THV chosen based on recent evidence showing lower gradients in valve-in-valve (ViV) subsets, when compared with other established self-expanding supra-annular THVs.^2^ Of 3 available sizes (23, 27, and 31 mm), 23 mm was selected based on the sizing guide and calculated in vivo average area of 272.2 mm^2^ & perimeter of 58.7 mm (see Figure 1, Figure 2, Figure 3).

## MANAGEMENT: REDO TAVI PROCEDURE

### Procedure

Primary arterial access was right femoral. The index THV stent frame was used as the marker for positioning. Invasive preprocedural PG and MG were 46 and 39 mm Hg, respectively. Predilatation was performed using an 18-mm noncompliant balloon, during rapid over-the-wire ventricular pacing (see Video 1). The ALLEGRA 23-mm THV was then delivered using the “Permaflow” delivery system aiming for an inflow to inflow position, allowing coverage of the nadir of the leaflets of the index THV while keeping the gold-markers of the ALLEGRA (marking the new valve plane) as close to the top of the commissure tab of the S3 as possible. This was done in the co-planar view (see Video 2).

**Figure 4:**
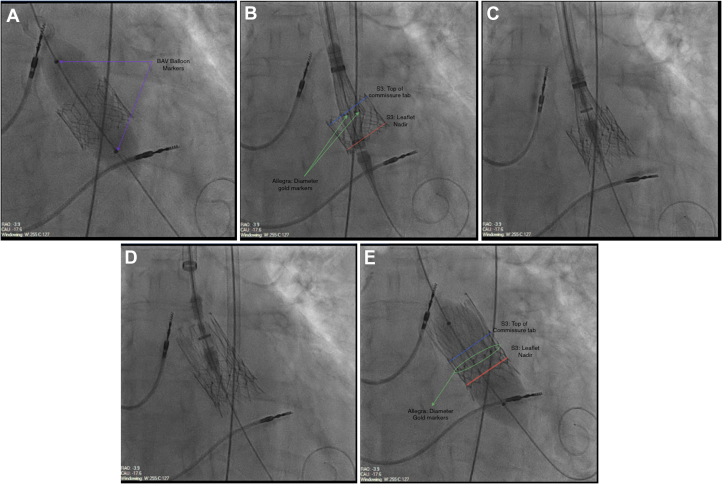
Procedure Steps (From top left): predilatation (A), positioning in “permaflow” (B), partial valve release (C), complete valve release (D), and post dilatation (E) (corresponding videos in Appendix 3).

Once optimal positioning was achieved, the valve was deployed in 2 further steps – inflow release and after assessment, final valve detachment (see Videos 3 and 4). No rhythm or conduction disturbances were noted, and hemodynamics remained stable. In order to maximize frame expansion, post dilatation was performed with a 20-mm non-compliant balloon (see Figure 4, Video 5). Postprocedural PG and MG were 10 and 9 mm Hg, respectively.

## Outcome and Follow-Up

Postprocedural echocardiography showed PG and MG of 21 and 11 mm Hg, respectively (lower than the gradients post index TAVI), with an improvement in LVEF to 45%. The patient was discharged home the next day and was stable on recent follow-up.

## Discussion

As guideline recommendations for TAVI have evolved to include younger patients, patient survival is increasingly exceeding THV durability.^3^ This has resulted in an increasing need for ViV TAVI procedures. We describe a patient who underwent redo TAVI using the ALLEGRA THV, with particular emphasis on the benefits of this device in this setting and the importance of meticulous preprocedural and periprocedural planning.

TAVI indications now include low surgical risk severe AS, severe bicuspid valve disease, and severe AR. Despite superior durability compared to surgical bioprostheses, increasing numbers of younger patients undergoing TAVI will likely result in more patients outliving their first THV.^1^^,^^3^^,^^4^ In addition, premature THV failure is also known to occur, resulting in earlier presentation for repeat intervention.^5^ In this case, with a native annulus area of 336 mm^2^, only a 20 mm balloon-expandable valve could be safely implanted, as a 23 mm valve would have resulted in 23% oversizing with an increased risk of annular injury. The downside of implanting such a small valve is suboptimal haemodynamics and in this case the postprocedural MG was already 17 mm Hg with an iEOA of 0.70 cm^2^/m^2^, consistent with moderate patient prosthesis mismatch. By definition, this represents bioprosthetic valve dysfunction and may translate, as in this case, into early bioprosthetic valve failure. It is interesting to note that despite the “Russian doll effect”, the MG following implantation of a 23 mm ALLEGRA THV was 11 mm Hg, lower than after the index procedure, while a similar iEOA of 0.70 cm^2^/m^2^ post Redo TAVI must be understood in context of LV function and flow (LVEF 60% vs 45%, SVI 39.6 vs 31 mL/m^2^, respectively, see Echocardiographic Summary in Supplement).

CT analysis is central to redo TAVI planning, allowing for valve selection and determining coronary obstruction risk. The Redo TAV app by Dr Bapat (see Figure 2) simplifies valve selection. The case we have described of an ALLEGRA in SAPIEN Redo TAVI underlines the importance of meticulous planning based on CT analysis.

As dedicated CT TAVI software become more sophisticated, with the advent of ViV & redo TAV processing packages, planning for these procedures will become more streamlined.

## Redo TAVI Planning

**Figure 5:**
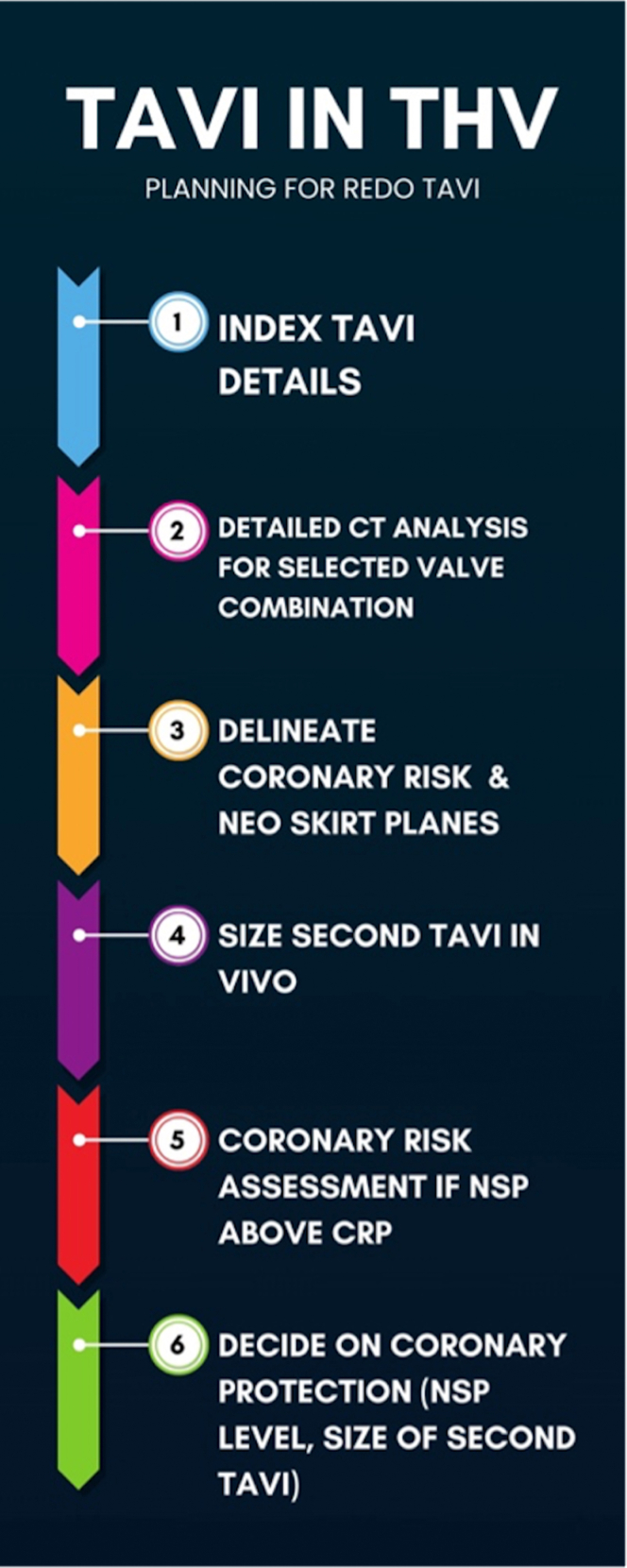
Transcatheter Aortic Valve Implantation (TAVI) in THV: Planning for Redo TAVI

Valve selection and implant strategy in redo TAVI aims to achieve the lowest possible gradients while minimizing the risk of coronary obstruction and maintaining future coronary access (See Figure 5). Coronary risk assessment involves identification of 2 planes and their relationship:

• Coronary Risk Plane (CRP): the plane at the base of the lowest patent coronary ostium
• Neoskirt Plane (NSP): the plane at the top of the “Neoskirt”

**Figure 6:**
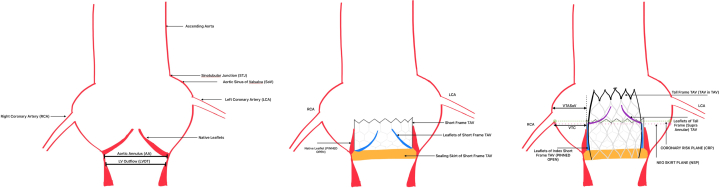
Schematic Illustrating Native Aortic Root Anatomy, Alterations Post Index TAVI, Alterations Post Redo TAVI With Positions of CRP and NSP

**Figure 7:**
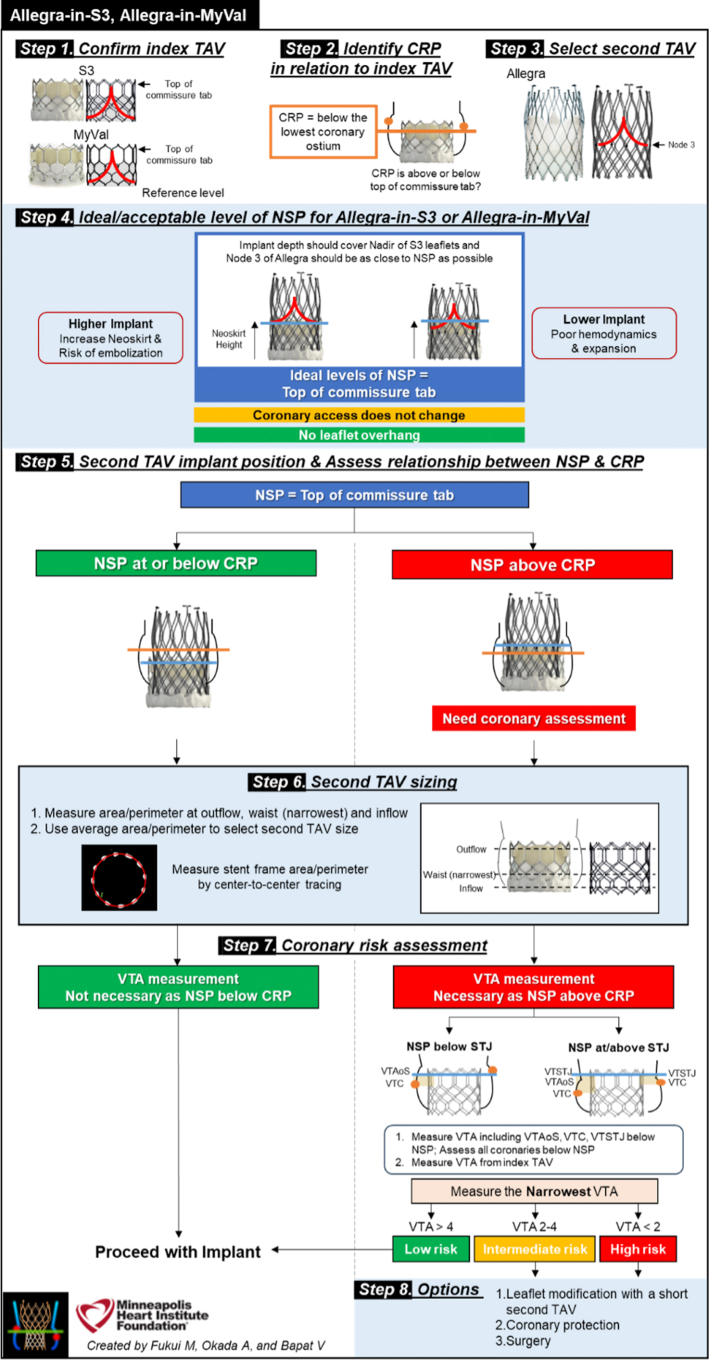
Redo TAV: ALLEGRA in S3—Planning (Reproduced With Permission From Dr Bapat)

**Figure 8:**
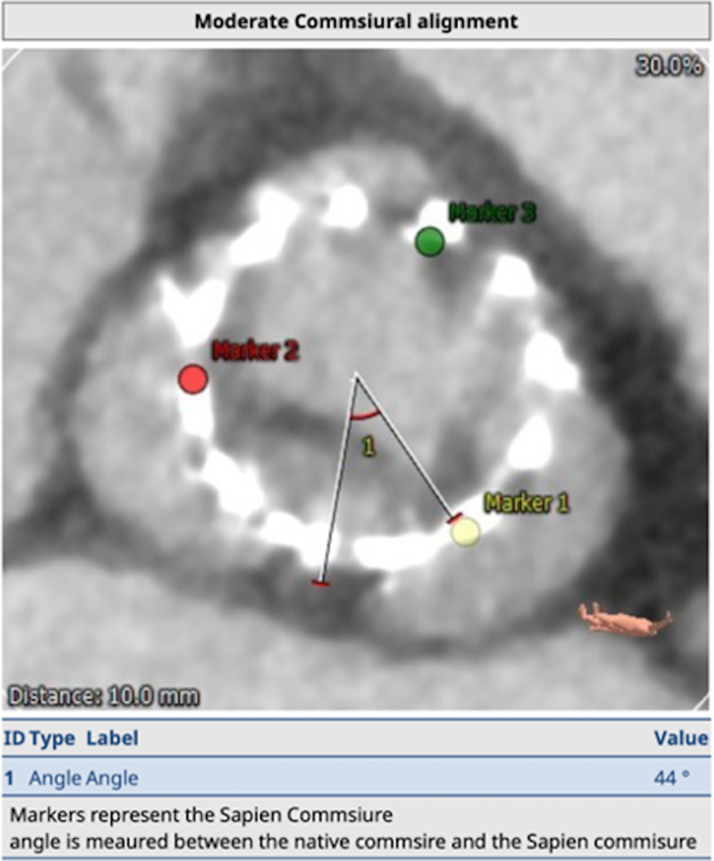
Moderately Misaligned Commissures of the Index Transcatheter Aortic Valve (Edwards S3 20)

**Figure 9:**
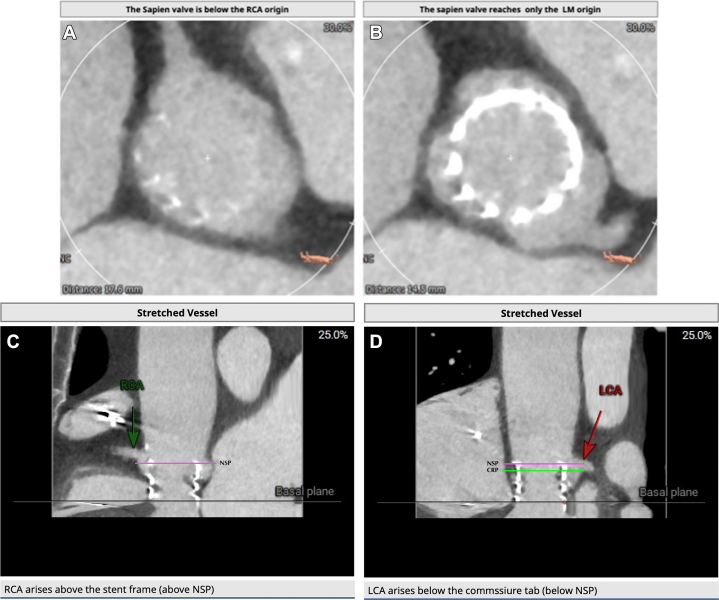
Relation of the Index Transcatheter Aortic Valve With Right (Right Coronary Artery [RCA]) and Left Coronary Artery (LCA)—Neoskirt and Coronary Risk Planes (A) Index THV and RCA, (B) Index THV and LCA, (C) RCA above NSP, (D) LCA (and CRP) below NSP.

The neoskirt is formed by the inner skirt of the second TAV in contact with the deflected open prosthetic leaflets of the Index TAV^6^ (see Figures 6 and 7).^6^ When the NSP is below the CRP, risk of coronary obstruction is low. If the NSP is above the CRP, coronary obstruction risk is increased and this risk must be defined (separately for both coronary arteries) by further measurements of “valve to aorta (VTA)” distances – these being between the outer larger valve of the redo TAV combination and the aorta. Depending on how much the NSP exceeds the CRP, VTA measurements also include a valve to coronary (VTC) distance or in case of the NSP being higher the sino-tubular junction (STJ), with potential for sinus sequestration, VTA measurements would involve VTC, valve to aortic Sinus (VTAoS) and valve to sino tubular Junction (VTSTJ) (see Central Illustration and Figures 6 and 7). Additionally, understanding the orientation of index THV “in vivo” to determine its commissural and coronary alignments is important for positioning of the second THV and to determine whether leaflet modification therapies to reduce coronary obstruction risks are required (see Figures 8 and 9).^6^

Short frame THV's can be re-treated with either short or tall THV's.^6^ We selected the supra-annular self-expanding ALLEGRA device based on observational data showing lower post procedure gradients compared to other balloon and self-expanding THV platforms in ViV subsets.^2^

In our case, the NSP of the combination (which would be at the top of the commissural tab of the index SAPIEN Valve),^6^ was below the right coronary artery, but above the left coronary artery (and CRP). However, it was below the STJ on the left and additional measurements of VTC and VTAoS (measured from the index in vivo TAV - Sapien 3 - in view of redo TAV with a self-expanding Allegra THV, with additional considerations for planned pre and post balloon aortic valvuloplasty) on the left were satisfactory, even considering dilatation with a 20 mm balloon which was thought to be the highest size balloon aortic valvuloplasty balloon that could be safely used (>4 mm is low risk^6^). Accordingly, we considered the risk of left coronary artery obstruction to be low (see Figure 9).

Redo TAVI planning can now be facilitated with the use of the Redo TAV app available on the Apple AppStore (see Figure 2) and Google PlayStore.

## The ALLEGRA Valve

The ALLEGRA THV is a self-expanding supra-annular valve with a stent frame that has concave and convex areas providing high EOAs even in small annuli (see Figure 3). It has evidence supporting its use in patients with severe AS.^7^^,^^8^ There is also data on its efficacy when used in ViV procedures for degenerated surgical bioprostheses.^2^^,^^9^ It has shown consistent performance and EOAs at lower implant depths compared to other THV platforms.^10^ The flexible outflow of the valve allows movement of the commissural posts with every cardiac cycle reducing leaflet stress. This mechanism may increase durability of the ALLEGRA THV; however, further evidence is awaited.

## Conclusions

As TAVI numbers rise, improving patient survival, younger age at index TAVI and the phenomenon of early THV failure, alone or in combination, are likely to increase indications for Redo TAVI procedures. Meticulous planning following CT analysis along with appropriate THV selection individualized to patient's anatomy are essential for procedural and hemodynamic success.

## Funding Support and Author Disclosures

The article processing charges, after publication acceptation by JACC, were made by Biosensors International Group Ltd. No other funding was received prior to or after this from Biosensors or any other organization.

**Take-Home Messages**

• Mastering CRP and NSP concepts is essential for understanding coronary access and obstruction risk during Redo-TAVI planning.
• The Redo-TAVI app (Dr Bapat), integrated with CT-based measurements, provides a systematic and simplified framework to select suitable THV options and optimize procedural strategy.
• The ALLEGRA THV, supported by data in valve-in-valve and redo-TAVI settings, offers favorable hemodynamics and potential durability advantages, especially in small annuli.

## Appendix

Video 1 Pre Dilatation

Video 2 Positioning in “Permaflow”

Video 3 Partial Valve Release

Video 4 Complete Valve Release

Video 5 Post Dilatation
