# Supplementary material for: Redo TAVI: Improved Hemodynamics With a Supra-Annular Valve in a Small Annulus
Source: JACC Case Rep. 2025 Dec 17;31(6):106501. doi: 10.1016/j.jaccas.2025.106501 (PMC12905691; doi:10.1016/j.jaccas.2025.106501)
Supplement: Supplemental Material [file mmc6.docx]

**[SUPPLMENTAL MATERIAL]**

**Appendix 1: Echocardiography summary**

Transthoracic Echocardiography (TTE) and Trans Oesophageal Echocardiography (TOE) findings.

|  | TTE post index  TAVI (02/03/21) | TTE (22/08/24) | TOE  (05/12/24) | TTE post  TAV-in-THV  (04/03/25) |
| --- | --- | --- | --- | --- |
| LVEF | 55-60% | 35% | 35-40% | 45% |
| Peak vel (m/s) | 2.70 | 3.37 | 3.24 | 2.31 |
| EOA ( cm²) | 1.05 | 1.1 | 1.1 | 1.05 |
| Indexed EOA  (cm²/m²) | 0.70 | 0.70 | 0.70 | 0.70 |
| Peak gradient mmHg | 29.2 | 45.5 | 42.0 | 21.3 |
| Mean gradient mmHg | 17.0 | 28.9 | 21.0 | 11.0 |
| Aortic regurgitation | Minimal AR | Moderate AR | Severe transvalvular AR | no significant residual AR |
| DVI | 0.46 | 0.44 | 0.38 | 0.37 |
| SVI  (ml/m^2^) | 39.6 | 55.0 | 46.7 | 31.0 |

Figure – TOE showing significant trans-valvular AR

**
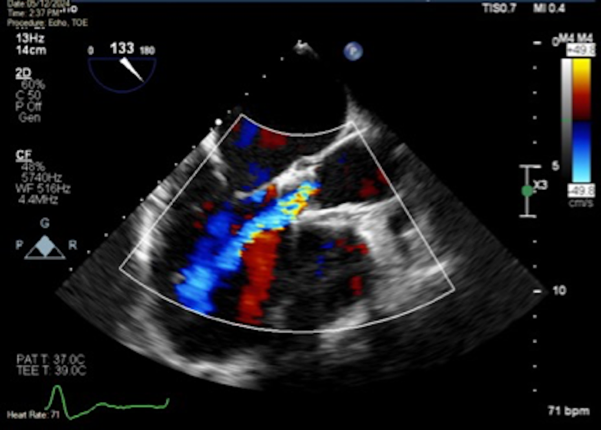

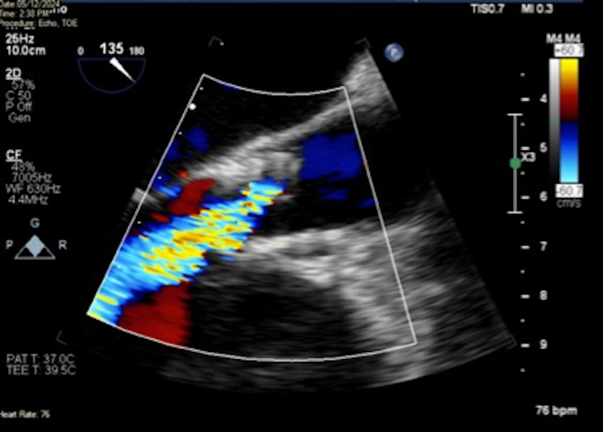
**

**Appendix 2 CT Measurements Summary**

Redo TAVI Measurements

The following are the key measurements from CT scan performed prior to redo TAVI. (Also see figures 1-3. Area measurements in figure 1 endeavored to be made through stent center of the blooming artifacts, as is recommended)


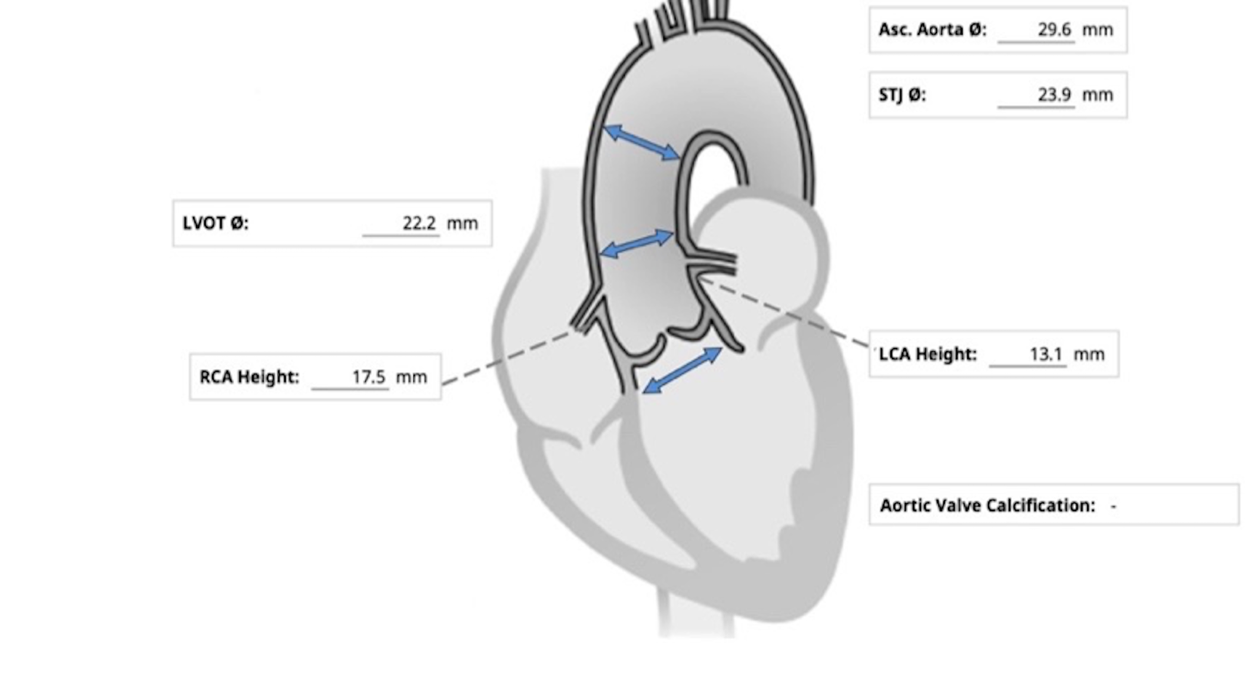


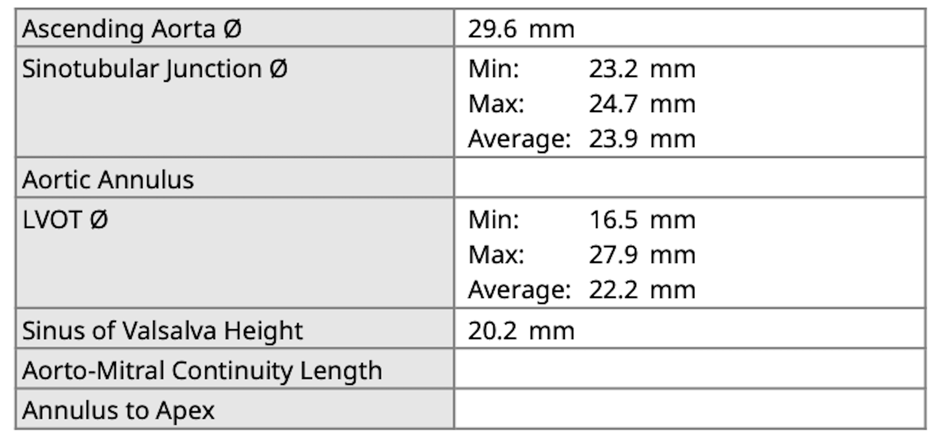


Index TAVI - Measurements

Analysis of the CT TAVI done on February 2021 on the 3mensio platform revealed the following measurements.

**AV and Root Measurements**

| Structure | Measurement | Value |
| --- | --- | --- |
| Aortic Annulus | Perimeter | 68.1 mm |
|  | Perimeter Derived Diameter (Ø) | 21.7 mm |
|  | Area | 336.4 mm² |
|  | Area Derived Diameter (Ø) | 20.7 mm |
|  | Min Diameter (Ø) | 16.8 mm |
|  | Max Diameter (Ø) | 25.6 mm |
|  | Average Diameter (Ø) | 21.2 mm |
|  | Eccentricity | 0.34 |
| LVOT | Diameter (Ø) | 19.9 mm |
|  | Min Diameter | 14.4 mm |
|  | Max Diameter | 25.5 mm |
| Ascending Aorta | Diameter (Ø) | 30.2 mm |
| Sinotubular Junction (STJ) | Diameter (Ø) | 24.8 mm |
| Coronary Heights | Left Coronary Artery (LCA) Height | 8.6 mm |
|  | Right Coronary Artery (RCA) Height | 8.9 mm |
| Sinus of Valsalva | Left Diameter (Ø) | 29.2 mm |
|  | Right Diameter (Ø) | 25.8 mm |
|  | Non-Coronary Diameter (Ø) | 27.8 mm |

**Appendix 3: Procedure Videos**

1. Video 1 Pre dilatation
2. Video 2 Positioning in “Permaflow”
3. Video 3 Partial valve release
4. Video 4 Complete valve release
5. Video 5 Post Dilatation
